# Patterns of differential gene expression in a cellular model of human islet development, and relationship to type 2 diabetes predisposition

**DOI:** 10.1007/s00125-018-4612-4

**Published:** 2018-04-19

**Authors:** Marta Perez-Alcantara, Christian Honoré, Agata Wesolowska-Andersen, Anna L. Gloyn, Mark I. McCarthy, Mattias Hansson, Nicola L. Beer, Martijn van de Bunt

**Affiliations:** 1 Wellcome Centre for Human Genetics, University of Oxford, Oxford, UK; 2 Department of Stem Cell Biology, Novo Nordisk A/S, Maaloev, Denmark; 3 Oxford Centre for Diabetes, Endocrinology & Metabolism, University of Oxford, Old Road, Oxford, OX3 7LE UK; 4 Oxford NIHR Biomedical Research Centre, Churchill Hospital, Oxford, UK; 5 Department of Stem Cell Research, Novo Nordisk A/S, Maaloev, Denmark

**Keywords:** Diabetes, Endocrine pancreas, In vitro differentiation, Islets, Stem cells, Transcriptome

## Abstract

**Aims/hypothesis:**

Most type 2 diabetes-associated genetic variants identified via genome-wide association studies (GWASs) appear to act via the pancreatic islet. Observed defects in insulin secretion could result from an impact of these variants on islet development and/or the function of mature islets. Most functional studies have focused on the latter, given limitations regarding access to human fetal islet tissue. Capitalising upon advances in in vitro differentiation, we characterised the transcriptomes of human induced pluripotent stem cell (iPSC) lines differentiated along the pancreatic endocrine lineage, and explored the contribution of altered islet development to the pathogenesis of type 2 diabetes.

**Methods:**

We performed whole-transcriptome RNA sequencing of human iPSC lines from three independent donors, at baseline and at seven subsequent stages during in vitro islet differentiation. Differentially expressed genes (*q* < 0.01, log_2_ fold change [FC] > 1) were assigned to the stages at which they were most markedly upregulated. We used these data to characterise upstream transcription factors directing different stages of development, and to explore the relationship between RNA expression profiles and genes mapping to type 2 diabetes GWAS signals.

**Results:**

We identified 9409 differentially expressed genes across all stages, including many known markers of islet development. Integration of differential expression data with information on transcription factor motifs highlighted the potential contribution of *REST* to islet development. Over 70% of genes mapping within type 2 diabetes-associated credible intervals showed peak differential expression during islet development, and type 2 diabetes GWAS loci of largest effect (including *TCF7L2*; log_2_FC = 1.2; *q =* 8.5 × 10^−10^) were notably enriched in genes differentially expressed at the posterior foregut stage (*q* = 0.002), as calculated by gene set enrichment analyses. In a complementary analysis of enrichment, genes differentially expressed in the final, beta-like cell stage of in vitro differentiation were significantly enriched (hypergeometric test, permuted *p* value *<*0.05) for genes within the credible intervals of type 2 diabetes GWAS loci.

**Conclusions/interpretation:**

The present study characterises RNA expression profiles during human islet differentiation, identifies potential transcriptional regulators of the differentiation process, and suggests that the inherited predisposition to type 2 diabetes is partly mediated through modulation of islet development.

**Data availability:**

Sequence data for this study has been deposited at the European Genome-phenome Archive (EGA), under accession number EGAS00001002721.

**Electronic supplementary material:**

The online version of this article (10.1007/s00125-018-4612-4) contains peer-reviewed but unedited supplementary material, which is available to authorised users.

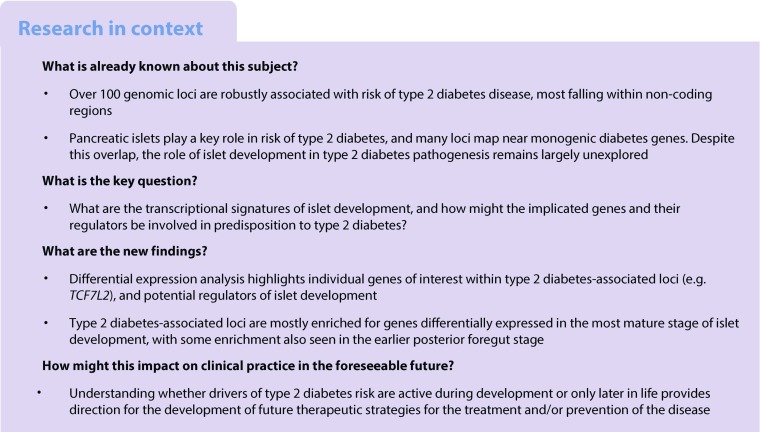



## Introduction

Our understanding of the genetic contribution to pathogenesis of type 2 diabetes has been greatly facilitated by genome-wide association studies (GWASs). These have identified over 100 genomic regions showing a robust association to disease risk [1]. However, teasing out the biological mechanisms underlying these disease associations continues to prove difficult, as most GWAS signals fall outside coding sequences. Broad inference across loci has been more successful, demonstrating from both phenotypic and genomic perspectives the importance of the pancreatic islet in risk of type 2 diabetes [2, 3].

Most functional follow-up of GWAS signals has involved studies in adult islets and/or a variety of beta cell lines, but there is mounting evidence that some of the implicated genetic variants influence islet development [4]. For example, many of the monogenic diabetes genes—most of which impact on islet development [5]—are also found in or near type 2 diabetes-associated loci [1]. Changes in the composition or number of islets as a result of events during development could lead to an altered functional islet mass in later life, increasing risk of type 2 diabetes.

Until recently, restricted access to human fetal material constrained the study of islet development to murine models. However, key differences between human and murine islet development [6], together with the potential of stem cell regenerative approaches to the treatment of diabetes, have motivated recent endeavours to differentiate human stem cells into pancreatic islet-like cells [7–9].

Islet differentiation protocols are rapidly improving [7, 10] and are now able to generate functional insulin-producing, although still somewhat immature, islet-like cells [8, 9]. In this study, we demonstrate how such cellular models of human pancreatic islet development can provide insights into the role of monogenic diabetes and type 2 diabetes-associated genes in islet development, and highlight the cellular pathways and mechanisms through which they act.

## Methods

### Generation of human induced pluripotent stem cells

Human induced pluripotent stem cell (iPSC) lines from three independent individuals without diabetes were obtained from the StemBANCC consortium (www.stembancc.org) (see ESM Methods). The generation of lines SB Ad2 and SB Ad3 has previously been described [10]. A third line, SB Neo1, was generated from commercial fibroblasts obtained from a neonatal donor of European descent with no reported diagnosis of diabetes (CC-2509, tissue acquisition number 15819; Lonza, Walkersville, MD, USA). Characterisation of all three lines has been reported elsewhere [10, 11]. All lines were free of mycoplasma.

### Ethics

All tissue samples for reprogramming were collected with full informed consent. Ethical approval for the StemBANCC study (UK) was received from the National Research Ethics Service South Central Hampshire A research ethics committee (REC 13/SC/0179).

### In vitro differentiation of iPSCs towards beta-like cells

The iPSC lines were cultured in mTeSR1 medium (StemCell Technologies, Vancouver, BC, Canada) at 37°C under 5% CO_2_, and passaged as single cells every 3–4 days or when confluent. In vitro differentiation involved the timely addition of recombinant growth factors and small molecules to sequentially generate cells representing key developmental stages of the endocrine pancreas: definitive endoderm, primitive gut tube, posterior foregut, pancreatic endoderm, endocrine progenitors, endocrine-like cells and beta-like cells. The differentiation protocol was carried out as described by Rezania and colleagues [9] with some modifications (ESM Tables 1, 2). All three iPSC lines were differentiated once, in parallel, using the same culture and differentiation media (ESM Methods).

### Flow cytometry

The efficiency of in vitro differentiation was evaluated by measuring the expression of stage-specific markers indicative of the development of the endocrine pancreas. For each specific stage, these were: definitive endoderm (SRY-box 17 [SOX17] and octamer-binding transcription factor 4 [OCT4, also known as POU5F1]); pancreatic endoderm (NK6 homeobox 1 [NKX6-1] and pancreas/duodenum homeobox protein 1 [PDX1]); and endocrine-like cells (NKX6-1, insulin [INS] and glucagon [GCG]) (ESM Fig. 1). Methods for flow cytometry were as previously described [10], and details of antibodies are listed in ESM Table 3.

### RNA extraction, sequencing and quantification

Cells were harvested and RNA extracted using TRIzol Reagent (ThermoFisher Scientific, Paisley, UK) as per the manufacturer’s guidelines. Library preparation and sequencing was performed at the Oxford Genomics Centre (Wellcome Centre for Human Genetics, Oxford, UK) as previously described [10]. RNA sequencing libraries were sequenced to a mean read depth of 148 (±12) million reads per sample. Reads were mapped to human genome build hg19, with GENCODE v19 (https://www.gencodegenes.org/releases/19.html) as the transcriptome reference, using STAR v.2.5 [12], followed by gene-level quantification with featureCounts from the Subread package v.1.5 (http://subread.sourceforge.net/) [13] (ESM Methods).

Principal component analysis was used to cluster samples with those from previously published studies [10, 14]. Correlation of gene expression patterns across all stages was calculated using the weighted gene co-expression network analysis (WGCNA) package (v.1.51) in R (v.3.3.2) (ESM Methods) [15, 16].

### Differential expression analysis

Analysis was performed on 15,221 autosomal protein-coding and long intergenic non-coding RNA (lincRNA) genes present in Ensembl Genes v88 (http://mar2017.archive.ensembl.org/index.html) with more than one count per million in all donors of at least one differentiation stage (ESM Table 4). Genes were normalised using the voom function within the limma package (v.3.32.5) in R [17]. The eBayes function in limma was used for differential expression analysis, comparing all the differentiation stages with iPSC as the baseline, and adjusting for donor effects. We adjusted *p* values for multiple testing (*q* values) using the Benjamini–Hochberg method [18].

To define stage-specific marker genes, differentially expressed genes (*q* < 0.01) with an absolute log_2_ fold change (FC) > 1 were assigned to the stage in which they were most upregulated compared with the baseline iPSC profile. When the log_2_FC was negative for all contrasted stages, the gene was assigned to iPSCs (ESM Table 5). For comparison with the previously reported protocol [10], published data were reprocessed in an analogous manner for the stages shared between the protocols (ESM Methods; ESM Tables 6, 7).

### Gene ontology and transcription factor binding motif enrichment

Differentially expressed genes in each stage were tested for enrichment in gene ontology terms for biological processes using the GOstats package (v. 2.40.0) in R [19]. All genes tested for differential expression were used as background. Significant gene ontology terms (*q* < 0.05) were retained (ESM Table 8).

For transcription factor enrichment, upstream regulators for the differentially expressed genes were predicted using the iRegulon (v. 1.3) Cytoscape plugin (ESM Methods) [20]. Motifs and chromatin immunoprecipitation (ChIP) sequencing tracks were ranked based on the normalised enrichment score (NES), with only those with an NES > 3 (corresponding to a false discovery rate (FDR) of 3–9%) being considered. Enriched motifs were then matched to transcription factors known to bind them (ESM Table 9).

### Type 2 diabetes and fasting glucose gene enrichment

Enrichment analysis was implemented in two ways: as a hypergeometric test in R (using all genes tested for differential expression as background) or using the gene-scoring function in MAGENTA [21] followed by a gene set enrichment analysis (GSEA) [22, 23] (ESM Methods).

For the hypergeometric test, we analysed the differentially expressed genes from each differentiation stage for enrichment in genes mapping to type 2 diabetes or fasting glucose GWAS signals, which were defined as protein-coding and lincRNA genes located within specified distance bins (0, 50, 100, 200 or 500 kb) surrounding the credible intervals for trait-associated loci. Credible intervals were defined by the boundaries of the 99% credible sets of variants [24] from DIAGRAM (96 loci) [25] and ENGAGE (16 loci) [26] consortium data, respectively (ESM Table 10). A subset of 15 loci was considered to influence type 2 diabetes via beta cell dysfunction; these loci included ones causing hyperglycaemia, reduced insulin processing and secretion, and reduced fasting proinsulin levels [27, 28] (ESM Table 11, ESM Methods).

For the analysis with MAGENTA and GSEA, we mapped SNPs from the type 2 diabetes GWAS meta-analysis from DIAGRAM (96 loci) [25], and the ranked list of *p* values for each gene was tested in GSEA (ESM Methods).

## Results and discussion

### Characterising an in vitro-derived model of human beta-like cells

To determine whether the differentiated cells followed normal islet development, we profiled gene expression patterns across iPSC and seven subsequent developmental stages in lines from three independent donors (SB Ad2, SB Ad3 and SB Neo1) differentiated in parallel. Each iPSC line successfully generated cells recapitulating key developmental stages of the endocrine pancreas as confirmed by the expression of known marker genes from developing and adult beta cells (ESM Fig. 2) [10].

**Fig. 1:**
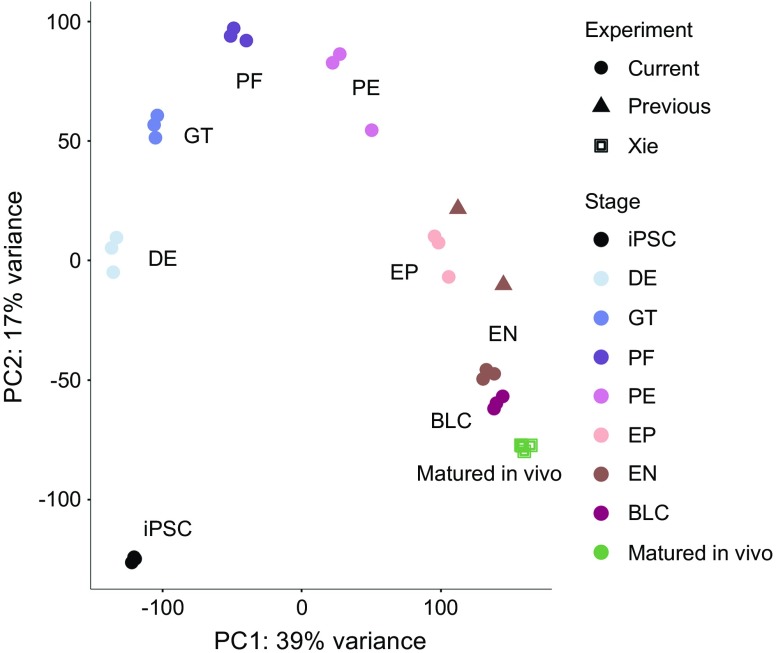
Principal component analysis of whole-transcriptome data derived from multiple differentiated human islet-like cell models. Data include all stages from our current differentiation protocol (Current), the most mature stage of a previously published differentiation protocol (Previous) [10], and cells derived via in vivo maturation by Xie and colleagues (Xie) [14]. The first two principal components (PC1, PC2) have been calculated using normalised gene counts for all stages of all three studies and corrected for batch effects. DE, definitive endoderm; GT, primitive gut tube; PF, posterior foregut; PE, pancreatic endoderm; EP, endocrine precursor; EN, endocrine-like cells; BLC, beta-like cells. Stages shown from the current study are iPSC, DE, GT, PF, PE, EP, EN and BLC. The stage shown from the previously reported study [10] is EN. The stage shown from Xie and colleagues’ in vivo maturation study [14] is ‘Matured in vivo’

Principal component analysis of the transcriptome showed that the beta-like cells generated in the current study clustered more closely with in vivo-matured islet-like cells [14] than cells from earlier differentiations [10] (Fig. 1, ESM Fig. 3). Differential expression analysis comparing transcriptomic profiles obtained from differentiations under current and previous protocols (see Methods) showed increasing divergence with differentiation stage (from 17 genes showing differential expression in iPSCs to 2095 at the endocrine-like cell stage) (ESM Table 7). Gene ontology analysis indicated that genes displaying increased expression at the endocrine-like cell stage (in comparisons of the current vs previous protocols) were enriched for terms including ‘regulation of insulin secretion’ (*q* = 2.3 × 10^−4^) and ‘hormone transport’ (*q* = 2.0 × 10^−5^).

Overall, cells generated in this study, compared with those previously reported [10], are more aligned to cells that have been further matured in vivo [14] (the current benchmark for most functionally mature endocrine pancreas-like cells). This reveals how advances in differentiation protocols are reflected in the transcriptome, particularly in the later stages of differentiation where there is a clear increase in the expression of genes essential for beta cell function and identity. This is the case for *MAFA*, which was completely absent in our previous differentiation protocol, and *INS*, whose high expression indicates the correct differentiation towards the last stage of beta cell development.

### Identifying transcriptional networks underlying islet development and diabetes

To characterise the transcriptomic landscape of each developmental stage in the in vitro-differentiated cells produced in this study, we assigned significantly differentially expressed genes to the stage at which they were most upregulated: if expression peaked in iPSCs, the gene was assigned to that stage (see Methods). We detected 9409 significantly differentially expressed genes (*q <* 0.01, absolute log_2_FC > 1) across all stages, ranging in number from 623 in the primitive gut tube stage to 2773 in iPSCs (ESM Table 5). Known developmental marker genes, such as *NEUROG3* in endocrine progenitors and *INS* in beta-like cells, were correctly assigned to their canonical stages. Gene ontology analysis of the sets of differentially expressed genes (ESM Table 8) showed enrichment in biological terms such as ‘hormone transport’ in endocrine-like cells (*q* = 0.047) and ‘regulation of insulin secretion’ in beta-like cells (*q* = 2.0 × 10^−4^).

The expression patterns of monogenic diabetes genes can point towards stages at which disruption of islet development has long-term consequences for glucose homeostasis. Of 28 genes implicated in monogenic or syndromic diabetes [1], 24 were differentially expressed in at least one stage of the in vitro-differentiated model. Nine mapped to the latest beta-like cell stage, but the other 15 showed significant upregulation earlier in differentiation (ESM Table 12). *GATA6*, for example, was differentially expressed at the definitive endoderm stage (log_2_FC = 9.5, *q* = 7.6 × 10^−11^), whereas *GATA4* was differentially expressed in posterior foregut cells (log_2_FC = 8.2, *q* = 1.9 × 10^−11^); the later expression of *GATA4* could contribute to the less severe phenotype of individuals carrying *GATA4* vs *GATA6* mutations [29, 30].

The differentiation model used in this study also sheds light on the developmental role of monogenic diabetes genes with lesser described roles. *LMNA*, for example, encodes a nuclear membrane protein involved in chromatin structure and nuclear stability; it has been implicated in the function and development of many tissues [31]. The diabetes in carriers of the *LMNA* mutation is mostly driven by altered adipose tissue deposition and insulin resistance [32]. However, the profile of *LMNA* expression during in vitro islet differentiation (peaking in pancreatic endoderm; log_2_FC = 1.1, *q =* 3.1 × 10^−3^) may indicate an additional impact on islet development [33].

The developmental competence of differentiating cells is in part driven by a subset of transcription factors that initiate and regulate changes in response to external stimuli, as highlighted by the many monogenic diabetes genes that are also transcription factors. To identify potential upstream transcriptional regulators active at each stage of islet development, we performed a WGCNA and determined the enrichment of transcription factor binding motifs and ChIP sequencing signals near differentially expressed genes using iRegulon (see Methods; ESM Table 9). This analysis confirmed the impact of well-established developmental transcriptional regulators such as the monogenic diabetes gene *HNF1B*, which showed iRegulon enrichment of its targets at the primitive gut tube stage (NES 3.0–5.7 [see Methods]). Some of these HNF1B targets also have known effects on pancreas development (*SMAD7* [34], *ID2* [35]), on mature islet function and on the development of other tissues that also arise from the gut tube (*GGCX*) [36].

Analysis of the sets of stage-specific differentially expressed genes also highlighted the targets of transcription factors with less-well studied roles in human islet development. For example, expression of the transcriptional repressor *REST* peaks in the intermediate steps of in vitro differentiation and declines at the endocrine-like cell and beta-like cell stages, with reciprocal expression patterns seen among its predicted targets. These targets include genes encoding neurexins (*NRXN1*, *NRXN2*) and subunits of the glutamate receptor channels *(GRIA1*, *GRIA2*, *GRID1*, *GRIK2*) implicated in insulin exocytosis [37, 38]. Correlation of gene expression with WGCNA assigns *REST* to the same cluster as *TCF7L2* and other genes from the Wnt signalling pathway, such as *TCF7*, *TCF3* and *TCF12* [39]. This pathway is important for islet development and is targeted in many in vitro differentiation protocols [8, 9]. These data therefore indicate that REST is likely to be an important transcriptional regulator of human islet development, both in intermediate (pancreatic endoderm, endocrine progenitor) and later (endocrine-like cell, beta-like cell) [40] stages of differentiation, as has also been recently suggested by studies in mice and humans [41, 42].

*TCF7L2* maps to the type 2 diabetes-associated locus with the largest common effect on disease risk [1]. Analysis of TCF7L2 targets (as assessed by ChIP sequencing with iRegulon) shows marked enrichment at the posterior foregut stage (NES = 3.4) that mirrors that of *TCF7L2* expression (log_2_FC = 1.2; *q =* 8.5 × 10^−10^). The expression of several other Wnt family members also peaks at the posterior foregut stage; these include the coactivator *CREBBP*, the binding sites of which are significantly enriched in type 2 diabetes-associated loci [43], and *HHEX*, which maps to a prominent type 2 diabetes-risk locus and is implicated in foregut development [44]. In the developing embryo, cells of the posterior foregut can differentiate into liver as well as endocrine pancreas [45]. Alleles associated with risk of type 2 diabetes within the *TCF7L2* and *HHEX* loci may influence early expression of these genes, which could affect development in multiple metabolic tissues. This view is supported by cellular and murine studies indicating that *TCF7L2* regulates beta cell development and function [46], including via indirect effects in supporting tissues [47], as well as affecting hepatic function [48]. Similarly, *Hhex* is essential for the differentiation of the posterior foregut into the liver in mice [44], yet is also thought to regulate delta cell identity and function in islets [49].

Thus, several key functional candidates mapping within type 2 diabetes GWAS signals, in addition to those which overlap known monogenic diabetes genes, appear to be active during this early critical window of pancreatic development. Studying these and other diabetes-relevant genes in stem cell-derived models can help to decipher the role of multiorgan developmental effects on pathogenesis of diabetes. By integrating the differential expression data with genomic annotations on transcription factor binding and clustering of longitudinal expression, we identified novel potential regulators orchestrating gene expression patterns within the different developmental stages. Such transcriptomic analysis can also illuminate the mechanisms of action for monogenic diabetes genes and inform the search for novel MODY genes that influence the same pathways.

### Developing and mature cells are enriched in genes within type 2 diabetes-associated loci

Most of the more than 100 type 2 diabetes susceptibility loci identified to date [1] map to non-coding regions of the genome and are likely to exert their effects through altered regulation of nearby genes. We examined the transcriptomic data for evidence of developmental stage-specific enrichment of genes near these loci.

**Fig. 2:**
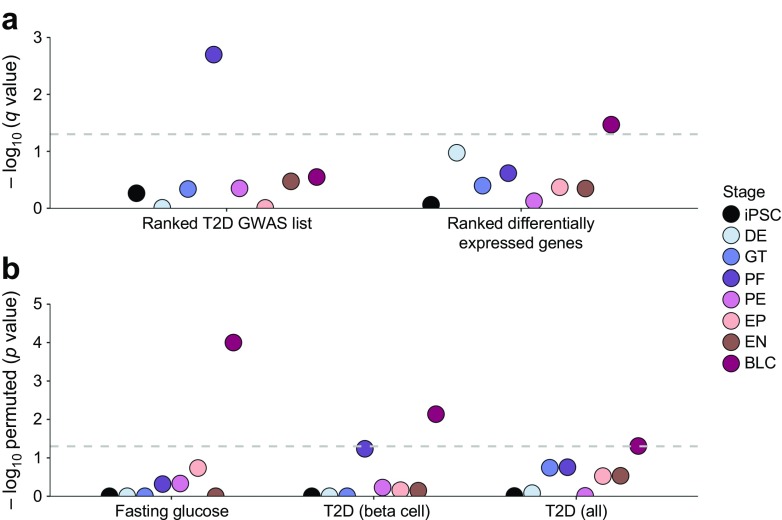
Both developing and mature islet-like cells are enriched for genes within type 2 diabetes-associated loci. (**a**) Results from the GSEA. SNPs from the type 2 diabetes GWAS meta-analysis from DIAGRAM (96 loci) [24] were mapped to genes, and type 2 diabetes association scores were calculated for each gene using MAGENTA. Two complementary analyses were performed: enrichment of all genes ordered by their MAGENTA scores in sets of differentially expressed genes for each stage (Ranked T2D GWAS list), and enrichment of differentially expressed genes per stage (ordered by *q* value) in significant (*p* < 0.05 by MAGENTA) gene scores (ranked differentially expressed genes). The *y*-axis represents the results of the GSEA in FDR-adjusted *p* values (*q* values, −log_10_). The horizontal grey dashed line marks the 5% significance threshold. (**b**) Results for the hypergeometric enrichment analysis. Enrichment was tested for all differentially expressed genes per stage in the 96 type 2 diabetes credible intervals [T2D (all)] from DIAGRAM [24] and the 16 fasting glucose credible intervals (Fasting glucose) from ENGAGE [25] (ESM Table 10), and for all differentially expressed genes in only physiological type 2 diabetes loci [T2D (beta cell)] (ESM Table 11). We consider beta cell function loci as 15 loci influencing hyperglycaemia, beta cell function and insulin processing [26, 27]. The *y*-axis represents the results of the hypergeometric test in permuted *p* values (−log_10_). The horizontal grey dashed line marks the 5% significance threshold. T2D, type 2 diabetes; DE, definitive endoderm; GT, primitive gut tube; PF, posterior foregut; PE, pancreatic endoderm; EP, endocrine precursor; EN, endocrine-like cells; BLC, beta-like cells

We first concentrated on genes whose coding sequence was at least partly contained within 99% credible intervals from type 2 diabetes GWAS fine-mapping efforts on the basis that these represented a set of genes likely to be substantially enriched for type 2 diabetes effector transcripts (see Methods). Of the 117 genes so defined, most (86; 73%) showed differential expression that peaked before the final beta-like cell stage (ESM Table 13); the stages of maximal differential expression were widely distributed. GSEA, which considers the strength of association at type 2 diabetes GWAS signals (see Methods), demonstrated enrichment of the type 2 diabetes GWAS loci with largest effect for differentially expressed genes at the posterior foregut stage (*q* = 0.002, Fig. 2a). This enrichment remained significant (*q* = 0.001) if the GWAS genes also implicated in monogenic diabetes (ESM Table 12) were excluded. Using a complementary GSEA approach that ranked the strength of differential expression of each gene (in *q* value) per stage, we compared the most differentially expressed genes at each stage for enrichment among type 2 diabetes GWAS loci; this analysis highlighted the beta-like cell stage (*q* = 0.033, Fig. 2a). This enrichment was no longer significant (*q* = 0.151) after monogenic diabetes genes had been excluded.

As an additional analytical approach, we performed a hypergeometric test for enrichment in the same set of 117 type 2 diabetes credible interval genes (see Methods). As opposed to the GSEA method above, this analysis does not consider the strength of differential expression (or of association with type 2 diabetes) above the significance threshold. This test again demonstrated that genes showing differential expression at the beta-like cell stage were enriched (compared with background) for location within type 2 diabetes credible intervals (permuted *p* value =0.049; Fig. 2b). Excluding the monogenic diabetes genes, and those that fell in the same credible interval, from the differentially expressed genes at each stage removed the significance of the beta-like cells (permuted *p* value =0.302). We repeated the enrichment test using a subset of 15 type 2 diabetes GWAS loci for which the evidence from physiological studies points most emphatically to risk of type 2 diabetes mediated via reduced insulin secretion (ESM Table 11) [27, 28]. In this analysis, enrichment for genes differentially expressed at the beta-like cell stage became more significant (permuted *p* value =0.007; Fig. 2b). This enrichment was reduced (but not eliminated; permuted *p* value =0.03) after excluding the monogenic diabetes genes and those within the same credible interval. Using the same approach of sampling from the hypergeometric distribution, we also detected enrichment for genes mapping to credible intervals for 16 loci significantly associated with fasting glucose (permuted *p* value =0.0002; Fig. 2b). Earlier stages of differentiation did not show significant enrichment for genes within type 2 diabetes or fasting glucose credible intervals. Nevertheless, the assignment of differentially expressed genes to a specific stage may lead to a wide distribution of signal that dilutes the power to detect significant enrichment at stages before the beta-like cell stage.

Type 2 diabetes-associated signals falling in non-coding regions have a presumed regulatory function: some may map to tissue-specific enhancers acting some distance away from their effector transcripts [50]. However, consistent with observations that most regulatory GWAS effects operate at relatively short distances [3], we found attenuation of these enrichment signals as we extended the analyses to include genes mapping at increasing distance from the credible intervals (see Methods), both for genes in all type 2 diabetes credible intervals and for the subset implicated in beta cell function (ESM Fig. 4).

The notable overlap between monogenic diabetes genes and those mapping within type 2 diabetes-associated loci supports the hypothesis that some component of type 2 diabetes susceptibility arises through impairment of islet development [1], concretely in the posterior foregut stage. The final stage in the islet development model (featuring cells expressing genes encoding the machinery to support glucose-stimulated insulin secretion) is also enriched for genes mapping to GWAS signals for both type 2 diabetes and fasting glucose. These data are consistent with the concept that type 2 diabetes-associated loci act both on the adult islet and during earlier developmental stages.

In summary, this study demonstrates how characterisation of gene expression during human islet differentiation can identify potential novel transcriptional regulators of the differentiation process, and provide insights into developmental aspects underlying inherited predisposition to type 2 diabetes. Further refinement of in vitro models of endocrine pancreas development will allow more detailed interrogation of the genes and pathways influencing islet development and function in humans. Mechanistic analyses of the contribution of candidate regulators of islet development to long-term islet function is enhanced by recent advances in clustered regularly interspaced short palindromic repeats- (CRISPR-) based approaches that allow their experimental manipulation in in vitro systems [51]. Stem cell-derived islets may also serve as a cost-effective platform for drug screening in research into treatment of diabetes, and could provide material for transplant into individuals with diabetes [8, 9].

## Electronic supplementary material

ESM (PDF 1364 kb)

ESM Table 4 (XLSX 2730 kb)

ESM Table 5 (XLSX 627 kb)

ESM Table 6 (XLSX 4224 kb)

ESM Table 7 (XLSX 295 kb)

ESM Table 8 (XLSX 45 kb)

ESM Table 9 (XLSX 206 kb)

ESM Table 10 (XLSX 16 kb)

## Data Availability

Sequence data have been deposited at the European Genome-phenome Archive (EGA), which is hosted by the European Bioinformatics Institute (EBI) and the Centre for Genomic Regulation (CRG), under accession number EGAS00001002721, and are also available on request from the authors.

## Abbreviations

FC: Fold change
GSEA: Gene set enrichment analysis
GWAS: Genome-wide association study
iPSC: Induced pluripotent stem cell
NES: Normalised enrichment score
NKX6-1: NK6 homeobox 1
WGCNA: Weighted gene co-expression network analysis
