# Supplementary material for: Patterns of differential gene expression in a cellular model of human islet development, and relationship to type 2 diabetes predisposition
Source: Diabetologia. 2018 Apr 19;61(7):1614–22. doi: 10.1007/s00125-018-4612-4 (PMC6354904; doi:10.1007/s00125-018-4612-4)
Supplement: Supplementary file 1 — (PDF 1364 kb) [file 125_2018_4612_MOESM1_ESM.pdf]

Electronic supplementary material (ESM)

ESM Methods

Generation of human induced pluripotent stem cells (hiPSCs)

Three hiPSC lines from individuals without diabetes were obtained from the StemBANCC consortium (www.stembancc.org), via the Human Biomaterials Resource Centre, University of Birmingham (http://www.birmingham.ac.uk/facilities/hbrc). Lines SB Ad2 and Ad3 were generated as described previously [10]. A third line, SB Neo, was generated from fibroblasts obtained from a commercial source (CC-2509, tissue acquisition number 15819; Lonza, Walkersville, MD, USA). The donor was neonatal, of European descent, with no reported diagnosis of diabetes. Detailed information on the reprogramming, culture, and characterisation of all three lines is reported elsewhere [10,11]. All lines were free of mycoplasma.

Ethics

All tissue samples for reprogramming were collected with full informed consent. Ethical approval for the StemBANCC study (UK) was received from the National Research Ethics Service South Central Hampshire A research ethics committee (REC 13/SC/0179).

In vitro iPSC differentiation towards beta-like cells

The iPSC lines were cultured in mTeSR1 medium (#05850; StemCell Technologies, Vancouver, BC, Canada) on tissue culture flasks coated with hESC-grade matrigel diluted per manufacturer's instructions (#354277; Corning, Bedford, MA, USA). Cells were maintained at 37°C, 5% CO_2 and passaged as single cells every 3-4 days or when confluent using TrypLE select (#12563011; ThermoFisher Scientific, Paisley, UK) and including 5µmol/l Rock Inhibitor (#Y0503; Sigma-Aldrich, St.Louis, MO, USA) in the medium the first day of passaging.

In vitro differentiation involved the timely addition of recombinant growth factors and small molecules to iPSCs, to sequentially generate cells representing key developmental stages of the endocrine pancreas: definitive endoderm (DE), primitive gut tube (GT), posterior foregut (PF), pancreatic endoderm (PE), endocrine progenitors (EP), endocrine-like cells (EN), beta-like cells (BLC). For differentiation, all three iPSC lines were harvested to a single cell suspension and seeded onto Corning CellBind surfaces coated with GFR-matrigel diluted 1:30 (#356230; Corning, Bedford, MA, USA). Cells were seeded at 350.000 cells/cm^2 in mTeSR1 medium with 5µmol/l Rock

inhibitor and incubated overnight at 37°C, 5% CO_2. After overnight incubation, medium was aspirated and cells were washed once in PBS (with Ca^{2+}/Mg^{2+}) before starting the differentiation. The differentiation protocol differed substantially to that used in our previous studies [10], and was carried out essentially as described by Rezania and colleagues [9] but with some modifications (**ESM Tables 1, 2**). All three iPSC lines were differentiated once, in parallel, using the same culture and differentiation media.

Flow cytometry

In vitro differentiation efficiency was evaluated by measuring the expression of stage-specific markers indicative of endocrine pancreas development. For each specific stage, these were: definitive endoderm (SRY-box 17 [SOX17] and octamer-binding transcription factor 4 [OCT4, also known as POU5F1]), pancreatic endoderm (NK6 homeobox 1 [NKX6-1] and pancreas/duodenum homeobox protein 1 [PDX1]) and endocrine-like cells (NKX6-1, insulin [INS] and glucagon [GCG]) (**ESM Figure 1)**. Methods for flow cytometry were as described previously [10], with details of antibodies listed in **ESM Table 3**.

RNA extraction, sequencing, and quantification

Cells were harvested into suspension using TrypLE Select before pelleting via centrifugation, and storage at -80°C in TRIzol reagent (15596–018; ThermoFisher Scientific, Paisley, UK). RNA was subsequently extracted as per manufacturer's guidelines. Library preparation and sequencing was performed at the Oxford Genomics Centre (Wellcome Centre for Human Genetics, University of Oxford) as described previously [10]. Briefly, polyadenylated transcripts were isolated with the NEBNext PolyA mRNA Magnetic Isolation Module (E7490 L; New England Biolabs, Ipswich, MA, USA), this followed by library preparation using the NEBNext Ultra Directional RNA Library Prep Kit for Illumina (E7420 L; New England Biolabs) with 12 cycles of PCR and custom 8 bp indexes. All libraries were multiplexed and sequenced with the TruSeq PE Cluster Generation Kit v3 and TruSeq SBS Kit v3 (PE-401-3001 and FC-401-3001 respectively; both Illumina, San Diego, CA, USA) over 7 lanes of Illumina HiSeq2000 as 100-nucleotide paired-end reads.

RNA-seq libraries were sequenced to a mean read depth of 148 (±12) million reads per sample. Reads were mapped to human genome build hg19, with GENCODE v19 (https://www.gencodegenes.org/releases/19.html) as the transcriptome reference, using STAR v.2.5 [4], followed by gene-level quantification with featureCounts from the Subread package v.1.5 (http://subread.sourceforge.net/) [5].

For the principal component analysis, we included samples differentiated with the current protocol, the previous protocol [10] and human embryonic stem cells (hESC) differentiated cells by Xie and colleagues (2013) [14]. The latter includes hESCs differentiated *in vitro* along the DE, GT, PF, PE, late PE and polyhormonal stages, and cells matured *in vivo* (in mice) from PE to functional endocrine cells. We retained genes detected in all three experiments, expressed at >1 count per million (cpm) in all donors of at least one stage in at least one of the experiments, resulting in 16,013 protein coding genes and lincRNAs. Counts were normalised and transformed to log-cpm using the *voom* function within the *limma* package (v.3.32.5) in R (v.3.3.2) [16, 17], and corrected for batch effects of the studies before principal component analysis using *removeBatchEffect* in *limma*.

Correlation of gene expression patterns across all stages was calculated using the weighted gene co-expression network analysis (WGCNA) package (v.1.51) in R [15]. Counts were normalized using variance stabilizing normalization and the network was constructed by hierarchical clustering of adjacency-based dissimilarity. This divided the genes into 29 co-expressed modules.

Differential expression analysis

Counts were filtered to include only genes that reached one cpm in all donors of at least one differentiation stage. Only autosomal protein-coding genes and long intergenic non-coding RNA (lincRNA) genes annotated in Ensembl Genes v88 (http://mar2017.archive.ensembl.org/index.html) were retained for downstream analysis. After removing genes with low counts, 15,221 protein coding genes and lincRNAs remained for normalization and differential expression analysis (**ESM table 4**). Gene counts were normalised and transformed to log-cpm using the *voom* function within the *limma* package (v.3.32.5) in R [16]. Contrasts were fitted comparing all the differentiation stages with iPSC as the baseline, adjusting for donor effect, as input for the eBayes function in *limma* for the differential expression analysis. All the reported *p*-values of differential expression are adjusted for false discovery rate (*q* values) using the Benjamini and Hochberg method [18]. To determine the stage-specific biomarkers, differentially-expressed genes ($q<0.01$) with absolute $\log_2$ fold change ($\log_2$FC)>1 were assigned to the stage in which they were most upregulated compared to the baseline iPSC profile. When the $\log_2$FC was negative for all contrasted stages, the gene was assigned to iPSC (**ESM Table 5**).

For the comparative analysis between the previous [10] and current protocols, we retained only genes detected in both experiments, expressed at >1 cpm in all donors of at least one stage in at least one of the experiments, resulting in 15,464 protein coding genes and lincRNAs (**ESM Table 6**). The differential expression analysis was performed exclusively for the stages shared between the protocols (iPSC, DE, GT, PF, PE and EN) (**ESM Table 7**). Of note, sample numbers differed between the protocols with three samples (SB Ad2.1, SB Ad3.1, SB Neo1.1) differentiated with the optimised protocol, and two samples (SB Ad2.1, SB Ad3.4) with the previously published protocol.

Gene ontology and transcription factor binding motif enrichment

Differentially expressed genes in each stage were tested for enrichment in gene ontology (GO) terms for biological processes, using the *GOstats* package (v. 2.40.0) in R [19]. All the genes tested for differential expression were used as background. GO terms that were significant (5%) after BH *p* value adjustment were retained (**ESM Table 8**).

For transcription factor enrichment, upstream regulators were predicted for the lists of differentially expressed genes assigned to each stage using the iRegulon (v1.3) Cytoscape plugin [20]. Significantly enriched binding sequence motifs were detected based on 1120 chromatin immunoprecipitation (ChIP) sequencing tracks from ENCODE and 9713 non-redundant transcription factor (TF) binding motifs from 7 species. Regions 10 kb around the transcription start site of each input gene were considered, and enrichment was run with default parameters. Motifs and ChIP sequencing tracks were ranked based on Normalized Enrichment Score (NES), with only those with NES>3 (corresponding to a false discovery rate (FDR) of 3–9%) being considered. Enriched motifs were then matched to transcription factors known to bind them (**ESM Table 9**).

Type 2 diabetes and fasting glucose gene enrichment

Enrichment analysis was implemented in two ways: as a hypergeometric test in R (using all genes tested for differential expression as background) or using the gene scoring function in MAGENTA [21] followed by a gene set enrichment analysis (GSEA) [22, 23].

For the hypergeometric test, we analysed the differentially expressed genes from each differentiation stage for enrichment in genes mapping to type 2 diabetes or fasting glucose GWAS signals, which were defined as protein coding genes and lincRNAs

located within 0, 50, 100, 200 or 500 kb surrounding the credible intervals for type 2 diabetes/fasting glucose-associated loci. Credible intervals were defined by the boundaries of the 99% credible sets of variants (that collectively encompass the 99% posterior probability of association with the trait) [24] from DIAGRAM (150,000 European subjects imputed to 1000 Genomes [25], 96 loci) and ENGAGE (46,694 European subjects imputed to 1000 Genomes [26], 16 loci), respectively (**ESM Table 10**). This resulted in lists of 195, 262, 340, 481 and 903 genes and lincRNAs present in the background for type 2 diabetes; and 14, 27, 39, 57 and 101 for FG.

We pooled as “beta-cell function” 15 loci influencing hyperglycaemia, beta-cell function (defined as reduced insulin secretion and fasting hyperglycaemia), and insulin processing (reduction in fasting proinsulin levels); or that were significantly associated in a GWAS for intravenous glucose test for insulin secretion [27, 28] (**ESM Table 11**).

The enrichment analysis by hypergeometric test in R was performed using all genes tested for differential expression as background. A distribution of *p* values was calculated for each stage from 10,000 random samplings (without replacement) of groups of genes, of the same size as the gene sets tested, from the background, which after applying the hypergeometric test gave a distribution of random *p* values. Then a permuted *p* value was calculated for each stage, defined as the fraction of *p* values from the random distribution that are more extreme (smaller) than the *p* value from the differentially expressed gene set for each stage.

For the analysis with MAGENTA and GSEA, we mapped SNPs from the type 2 diabetes GWAS meta-analysis from DIAGRAM (96 loci) [25] and generated the ranked list of *p* values for each gene using 10,000 permutations and gene boundaries of 110 kb upstream and 40 kb downstream each transcript. This gene score list was tested in GSEA for enrichment using 1,000 permutations, in two ways: for enrichment of the complete gene score list in sets of differentially expressed genes for each stage, or conversely for enrichment of these stages in significant gene scores ($p<0.05$ by MAGENTA). All the reported *p* values for GSEA are adjusted for false discovery rate (*q* values).

ESM Tables and Excel table legends

ESM Table 1. Overview of media used in differentiation.

Stage	Days	Medium	Factors	Conc.	Vendor	Catalog #
Definitive endoderm	1	MCDB131-1	CHIR99021	3µmol/l	Axon, Groningen The Netherlands.	1386
			Activin A	100ng/ml	Peprotech, Rocky Hill, NJ, USA.	120-14
	1	MCDB131-1	CHIR99021	0.3µmol/l	Axon	1386
			Activin A	100ng/ml	Peprotech	120-14
	1	MCDB131-1	Activin A	100ng/ml	Peprotech	120-14
Primitive gut tube	2	MCDB131-1	KGF	50ng/ml	Peprotech	100-19
			Ascorbic acid	0.25mmol/l	Sigma-Aldrich	A4544
Posterior foregut	2	MCDB131-2	Retinoic acid	1µmol/l	Sigma-Aldrich	R2625
			Sant-1	0.25µmol/l	Sigma-Aldrich	S4572
			KGF	50ng/ml	Peprotech	100-19
			LDN	100nmol/l	Stemgent, Cambridge, MA, USA	04-0074
			PKC act V (TBP)	200nmol/l	Merk, Darmstadt, Germany	565740
			Ascorbic acid	0.25mmol/l	Sigma-Aldrich	A4544
Pancreatic endoderm	3	MCDB131-2	Retinoic acid	0.1µmol/l	Sigma-Aldrich	R2625
			Sant-1	0.25µmol/l	Sigma-Aldrich	S4572
			LDN	200nmol/l	Stemgent	04-0074
			PKC act V (TBP)	100nmol/l	Merk	565740
			KGF	2ng/ml	Peprotech	100-19
			Ascorbic acid	0.25mmol/l	Sigma-Aldrich	A4544
Endocrine progenitors	3	MCDB131-3	Retinoic acid	0.05µmol/l	Sigma-Aldrich	R2625
			Sant-1	0.25µmol/l	Sigma-Aldrich	S4572
			LDN	100nmol/l	Stemgent	04-0074
			Alk5i II	10µmol/l	Enzo	ALX-270-445
			T3	1µmol/l	Sigma-Aldrich	T6397
			Heparin	10ug/ml	Sigma-Aldrich	H3149
Endocrine-like cells	7	MCDB131-3	LDN	100nmol/l	Stemgent	04-0074
			T3	1µmol/l	Sigma-Aldrich	T6397
			Alk5i II	10µmol/l	Enzo	ALX-270-445
			γ-sec. inh XX	100nmol/l	Merk	565789
			Heparin	10ug/ml	Sigma-Aldrich	H3149
Beta-like cells	7	MCDB131-3	T3	1µmol/l	Sigma-Aldrich	T6397
			Alk5i II	10µmol/l	Enzo	ALX-270-445
			N-acetyl-Cys.	1mmol/l	Sigma-Aldrich	A9165
			Trolox	10µmol/l	Merk	648471
			R428	2µmol/l	SelleckChem, Houston, TX, USA	S2841
			Heparin	10ug/ml	Sigma-Aldrich	H3149

ESM Table 2. Growth factor formulation for pancreas differentiation.

Stage 1-2	Stage 3-4	Stage 5-7	Catalogue #
MCDB131	MCDB131	MCDB131	ThermoFisher 10372-019
0.1% P/S	0.1% P/S	0.1% P/S	ThermoFisher 15140-122
1.5g/l NaHCO3	2.5g/l NaHCO3	1.5g/l NaHCO3	ThermoFisher 25080-060
1 x Glutamax	1 x Glutamax	1 x Glutamax	ThermoFisher 35050-038
10mmol/l Glucose final	10mmol/l Glucose	20mmol/l Glucose	Fisher Scientific D16500
0.5% BSA	2% BSA	2% BSA	Proliant (Ankeny, IA, USA) 68700
	1:200 ITS-X	1:200 ITS-X	ThermoFisher 51500-056
		10μmol/l Zinc sulfate	Sigma-Aldrich Z0251

ESM Table 3. Antibodies used for flow cytometry.

Antigen	Conjugate	Vendor	Catalogue #	Dilution
C-Peptide	Alexa Fluor 647	BD Biosciences (Franklin Lakes, NJ, USA)	565831	1:100
Glucagon	PE	BD Biosciences	565860	1:40
NKX6.1	Alexa Fluor 647	BD Biosciences	563338	1:40
Oct3/4	Alexa Fluor 647	BD Biosciences	560329	1:10
PDX1	Alexa Fluor 488	BD Biosciences	562274	1:40
Sox17	Alexa Fluor 488	BD Biosciences	562205	1:40

Antibodies were validated in all cases for absence of staining in developmental stages where they are not expressed.

ESM Table 4. Gene counts from each stage and sample, generated with the current differentiation protocol.

ESM Table 5. Results of the differential expression analysis contrasting the expression of each gene at each stage against the iPSC baseline from the current differentiation protocol.

ESM Table 6. Gene counts from each stage and sample generated with the current and previously-published [10] differentiation protocols.

ESM Table 7. Results of the differential expression analysis comparing corresponding stages (iPSC, DE, GT, PF, PE and EN) between the current and previously-published [10] differentiation protocols.

ESM Table 8. Enrichment in gene ontology (GO) terms for biological processes of differentially expressed genes in each stage.

ESM Table 9. Predicted transcription factors with iRegulon from enriched transcription factor binding motifs and ChIP-seq tracks from the differentially expressed genes in each stage.

ESM Table 10. 99% credible intervals of 96 type 2 diabetes-associated loci (from DIAGRAM) and 16 fasting glucose-associated loci (from ENGAGE).

ESM Table 4 to 10 are available as a separate Excel file.

ESM Table 11. Beta cell function loci, as defined by: i) Dimas and colleagues (2014) [26] as involved in hyperglycaemia (HG), beta cell function (BC) or insulin processing (PI), or 2) Wood and colleagues' (2017) [27] following GWAS of insulin secretion after intravenous glucose tolerance test. UC: uncharacterized.

Locus	Dimas *et al.*, 2014	Wood *et al.*, 2017 (Genome wide)
ADCY5	BC	Yes
ARAP1	PI	Yes
CDKAL1	BC	Yes
CDKN2A_B	BC	Yes
DGKB	BC	-
GCK	HG	-
HHEX_IDE	BC	-
HNF1A	UC	Yes
IGF2BP2	UC	Yes
*KCNQ1*_rs163184	UC	Yes
MTNR1B	HG	Yes
PROX1	BC	-
SLC30A8	BC	Yes
TCF7L2	BC	Yes
THADA	BC	-

ESM Table 12. Differentially expressed genes identified as causal for monogenic diabetes by Fuchsberger *et al.* (2016) [1].

iPSC	DE	GT	PF	PE	EP	EN	BLC
ALMS1	*GATA6*	*PPARG*	*GATA4*	*HNF4A*	*RFX6*	*PDX1*	*ABCC8*
		HNF1B		*PTF1A*	*MNX1*	*NEUROD1*	*KCNJ11*
				INSR	*UCP2*		*WFS1*
				LMNA	*NEUROG3*		*HNF1A*
							GCK
							GLIS3
							INS
							HADH
							SLC2A2

ESM Table 13. Differentially expressed protein-coding genes and lincRNAs within T2D-associated loci credible intervals (0 kb distance).

iPSC	DE	GT	PF	PE	EP	EN	BLC
POU5F1	*DMRTA2*	*PPARG*	*TCF7L2*	*HNF4A*	*CALCOCO2*	*ZZEF1*	*ABCC8*
GATAD2A	*RFXANK*	*FGFR3*	*HHEX*	*PROX1*	*TP53INP1*	*CMIP*	*KCNJ11*
HMG20A	*RBMS1*	*NRXN3*	*MAU2*	*LTBP3*	*TTLL6*	*IDUA*	*WFS1*
TCF19	*MYL5*	*LAMA1*	*FAM89B*	*HKDC1*	*MAN2A2*	*NCAN*	*HNF1A*
ZNF101	*RNF11*	*FGFRL1*	*ACSL1*		*PDE6B*	*KLHL42*	*GCK*
PABPC4	*HMGA2*	*SPON2*	*PCGF3*		*CDKN1C*	*GIP*	*GLIS3*
TACC3	*CDKN2C*	*ZMIZ1*	*LPAR2*		*ANK1*	*PAM*	*CAMK2B*
ATP5G1	*APOE*	*RILPL2*	*FCHSD2*		*CRIPAK*	*ATG16L2*	*STARD10*
SIPA1	*PTPRD*	*MFSD7*	*MALAT1*		*NKX6-3*	*GIPR*	*FITM2*
ZFAND6	*APOC1*	*DPY19L4*	*DGAT1*		*CDKAL1*	*BMP8A*	*FBXW7*
CENPW	*HLA-DRB1*		*ARL15*		*YJEFN3*	*CDKN2B*	*GDAP1L1*
CDK2AP1					*EHBP1L1*	*ARL6IP4*	*CYB5D2*
HSD17B12							*NUCB2*
PEAK1							*EML2*
PRC1							*TMEM175*
ATP5I							*VPS13C*
SSSCA1							*MXD4*
RCCD1							*SLC30A8*
NR2C2AP							*MTMR3*
BCAR1							*TTC39A*
UBE2E2							*PITPNM2*
CILP2							*NAT8L*
RNF212							*NCR3LG1*
LINC00491							*CPLX1*
C5orf30							*PLEKHA1*
							ABCB9
							MTNR1B
							DGKB
							C2CD4A
							SLC26A1
							C4orf48

ESM Figures

ESM Figure 1.

a

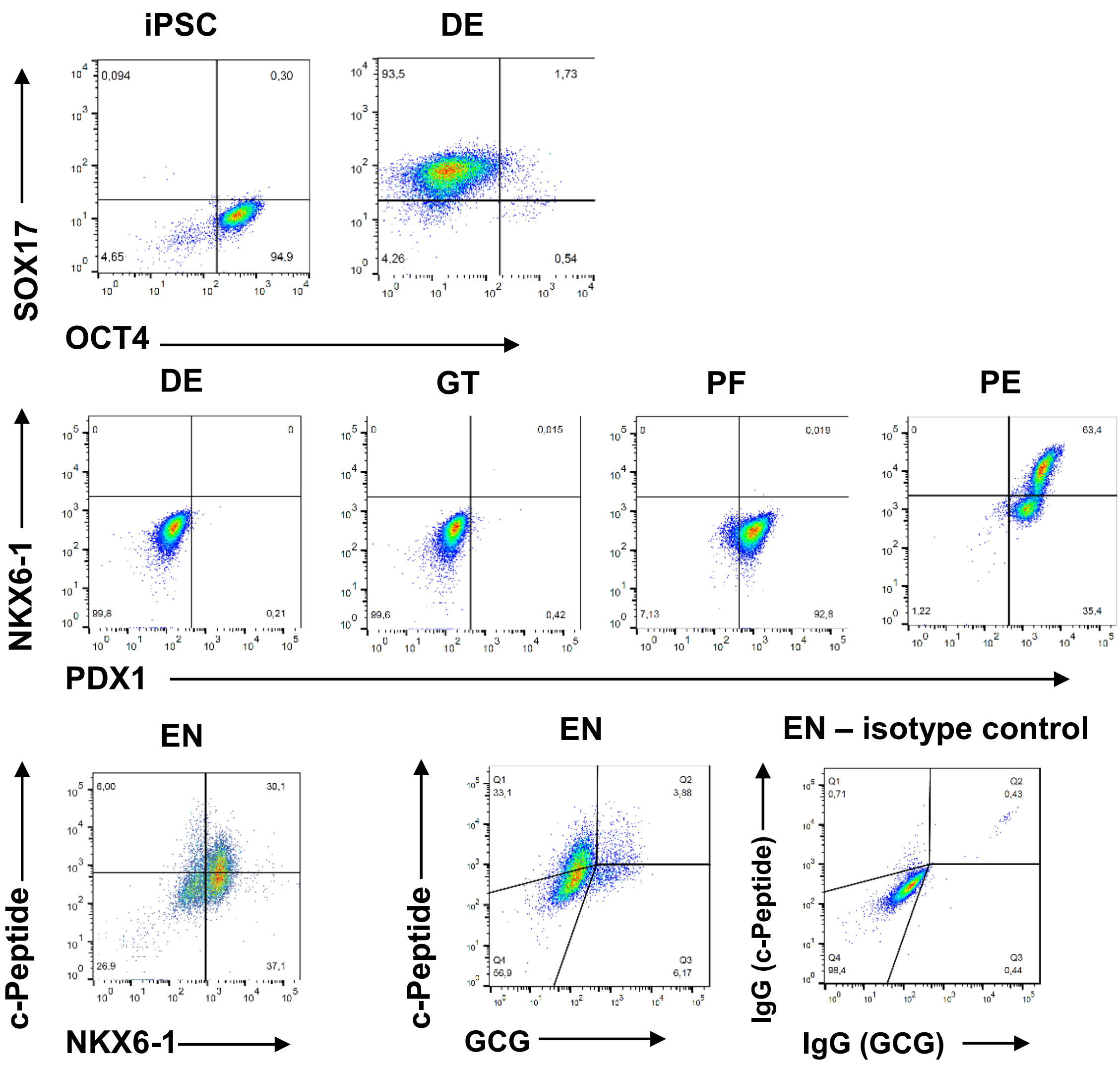

b

Samples	DE		PE		EN	
	Sox17+	Oct4+	PDX1+	PDX1/NKX6-1+	c-Peptide/NKX6-1+	c-Peptide/GCG+
**SB Ad2**	98.31%	1.29%	89.83%	55.70%	N/A	N/A
**SB Ad3**	95.23%	2.27%	92.82%	63.40%	30.10%	3.88%
**SB Neo1**	91.97%	0.38%	87.70%	35.70%	30.00%	11.00%

ESM Figure 1. Evaluating *in vitro* differentiation efficiency of human islet-like cells. a) Example FACS analysis of markers of the definitive endoderm, pancreatic endoderm and endocrine-like cells stage for SB Ad3 cells. In iPSC through PE the gating strategy was based on negative control cells for each marker, while for the EN stage we base our gating on the isotype control, which represents unspecific binding of the secondary antibodies. iPSC: induced pluripotent stem cells; DE: definitive endoderm; GT: primitive gut tube; PF: posterior foregut; PE: pancreatic endoderm; EN: endocrine-like cells. b) Percentage of cells expressing known developmental marker genes. No data available for SB Ad2 at EN stage due to limited cell numbers. c-Peptide is a surrogate for insulin.

ESM Figure 2.

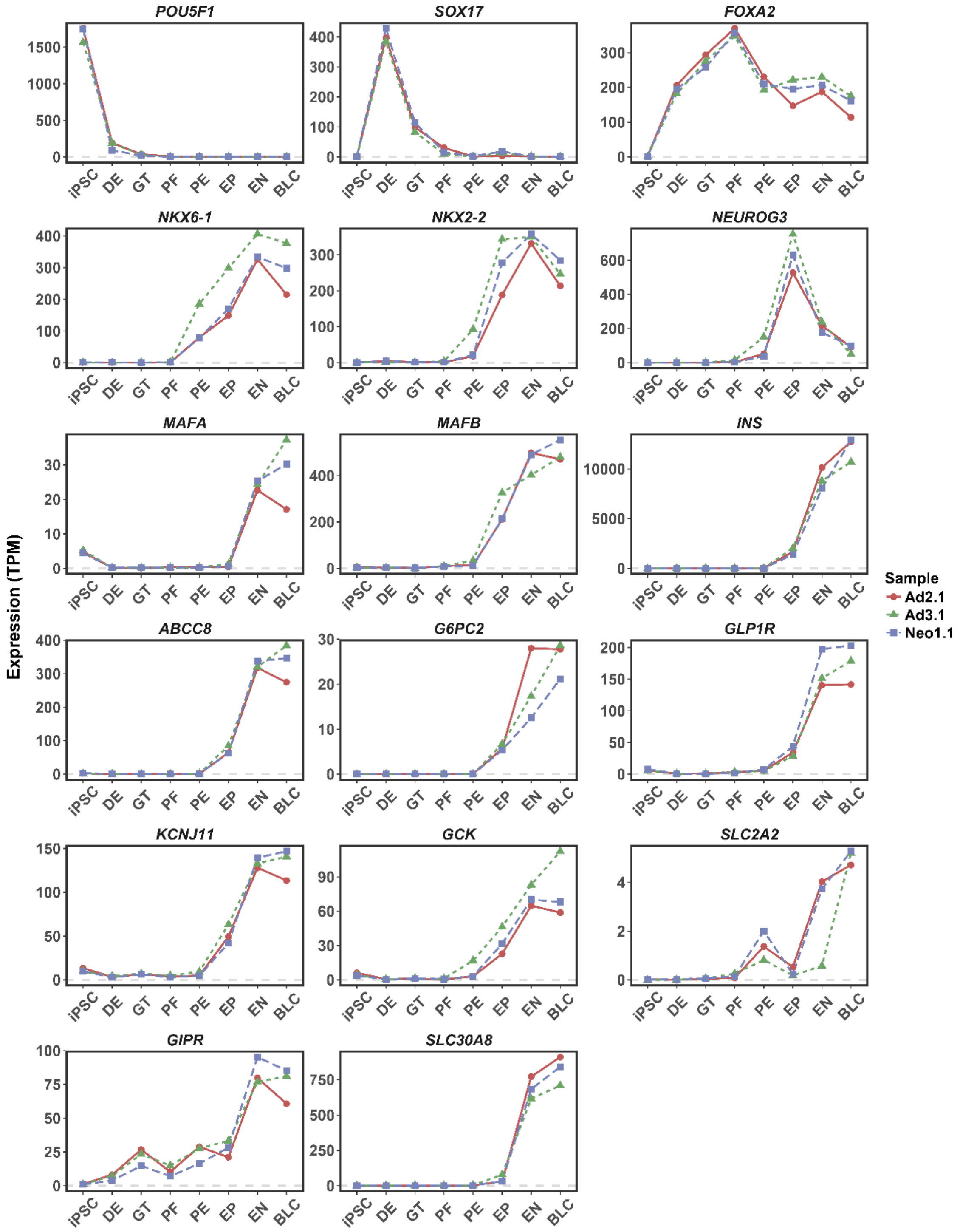

ESM Figure 2. Expression patterns (in transcripts per million) of islet development and cell marker genes of endocrine cells: *POU5F1* (iPSC), *SOX17* (DE), *FOXA2* (DE onwards), *PDX1* (PF onwards), *NKX6-1* (PE onwards), *NEUROG3* (EP), *MAFA* and *INS* (EN onwards), and additional beta cell markers, follow the expected patterns of expression. iPSC: induced pluripotent stem cells; DE: definitive endoderm; GT: primitive gut tube; PF: posterior foregut; PE: pancreatic endoderm; EP: endocrine precursor; EN: endocrine-like cells; BLC: beta-like cells.

ESM Figure 3.

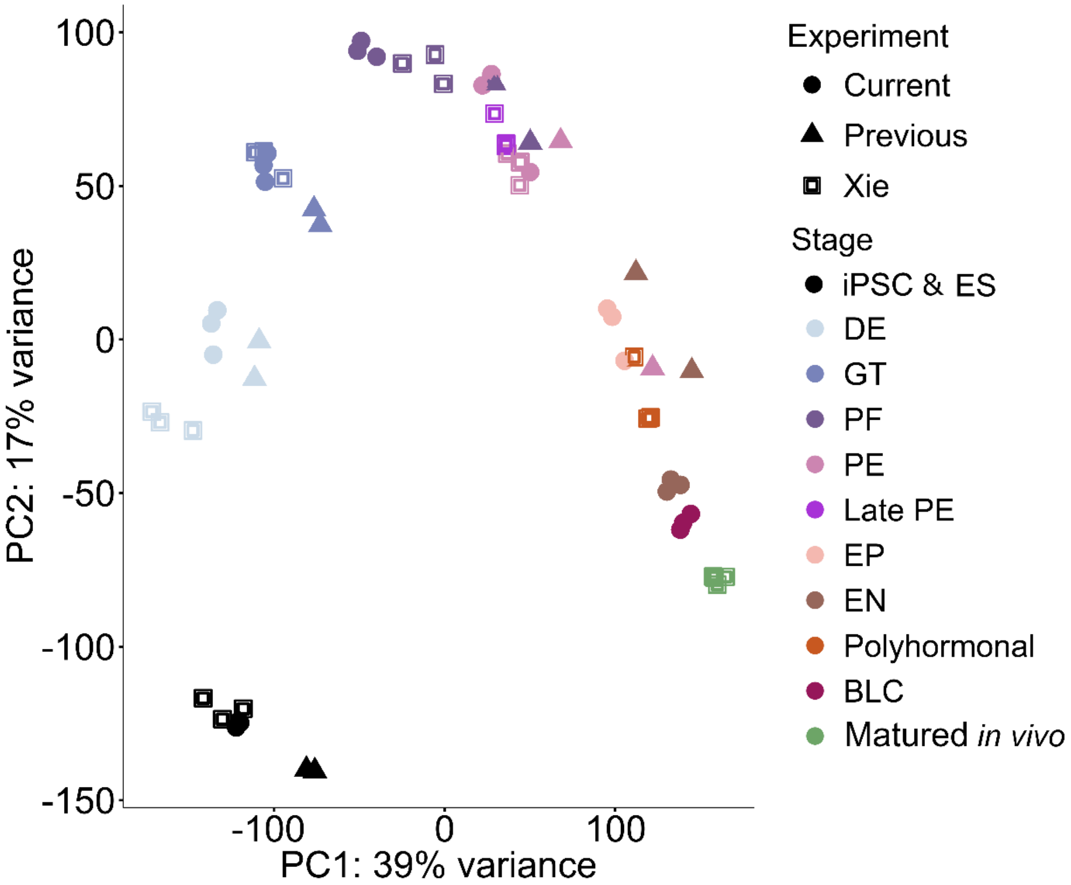

ESM Figure 3. Principal component analysis of whole transcriptome data derived from multiple differentiated human islet-like cell models. Plotted are differentiated cell samples generated using our current ("current") and previously-reported [10] differentiation protocol ("previous"), and cells derived via *in vivo* maturation by Xie and colleagues ("Xie") [14]. Shown are the first two principal components of normalized gene counts for all stages of the three studies, corrected for batch effects. iPSC: induced pluripotent stem cells; ES: embryonic stem cells; DE: definitive endoderm; GT: primitive gut tube; PF: posterior foregut; PE: pancreatic endoderm; EP: endocrine precursor EN: endocrine-like cells; BLC: beta-like cells. Stages included in the current study: iPSC, DE, GT, PF, PE, EP, EN, BLC. Stages in previously-reported study [10]: iPSC, DE, GT, PF, PE, EN. Stages from Xie and colleagues' *in vivo* maturation study [14]*:* ES, DE, GT, PF, PE, late PE, polyhormonal, matured *in vivo*.

ESM Figure 4.

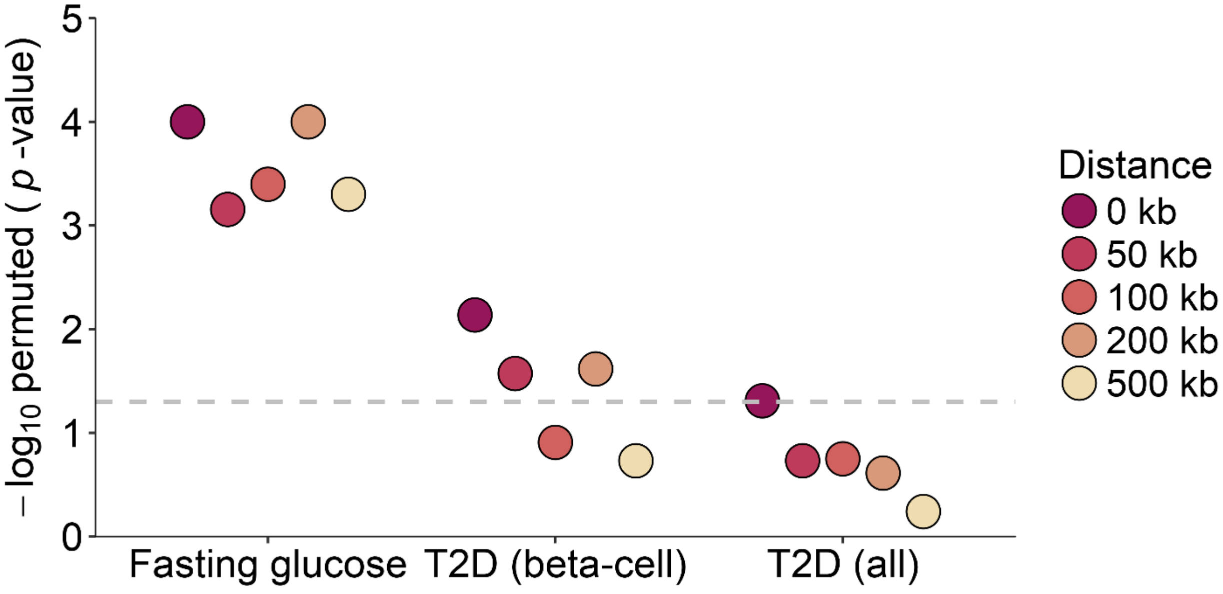

ESM Figure 4. Enrichment results in genes inside variable distances around the type 2 diabetes and fasting glucose credible regions, for differentially expressed genes assigned to the BLC stage. Enrichment was tested for all differentially expressed genes in the 96 type 2 diabetes credible intervals ("T2D [all]") from DIAGRAM [24] and the 16 fasting glucose credible intervals ("Fasting glucose") from ENGAGE [25] (ESM Table 10), and for all differentially expressed genes in only physiological type 2 diabetes loci ("T2D [beta-cell])", ESM Table 11). We consider beta cell function loci 15 loci influencing hyperglycaemia, beta-cell function, and insulin processing [26, 27]. The y-axis represents the results of the hypergeometric test in permuted *p* values (-log10). The horizontal grey dashed line marks the 5% significance threshold.
